# Currents status of radiotracers for breast cancer imaging in PET

**DOI:** 10.1016/j.tranon.2025.102304

**Published:** 2025-02-07

**Authors:** Chloé Jean, Stéphane Roux, Abdelilah Aziz, Maxence Mocquery-corre, Rana Bazzi, Yacine Merrouche, Stéphane Dedieu, Nicolas Etique, Dimitri Papathanassiou, Jérôme Devy

**Affiliations:** a Université de Reims Champagne Ardenne, UMR CNRS 7369 MEDyC, Reims, France; b Institut Godinot, Reims, France; c Université de Reims Champagne Ardenne, CRESTIC, Reims, France; d Université Marie et Louis Pasteur, CNRS, Chrono-environnement (UMR 6249), F-25000 Besançon, France; e Université de Reims Champagne Ardenne, UFR de Médecine, France

**Keywords:** Radiotracers, Breast cancer, Radiopharmaceuticals, Targeted radionuclide therapy, Nanoparticles

## Abstract

Radiolabeled molecules have become valuable tools in the diagnosis, monitoring, and treatment of cancer, particularly breast cancer. Through the use of radiotracers, clinicians can target specific tumor cells, assess microenvironments, and identify metastases. These radiopharmaceuticals, based on radionuclides, enable both imaging and therapeutic applications, leading to personalized cancer treatment. Techniques such as PET, SPECT, and the use of nanoparticles for theranostics are at the forefront of innovation, offering improved precision in both diagnosis and therapy. This review explores the various ways in which radiotracers are leveraged in modern oncology, with a focus on breast cancer, and highlights recent advancements in targeted radionuclide therapy and nanoparticle-based applications.

## Highlights

• Radiolabeled molecules play a crucial role in cancer diagnosis and therapy, particularly in breast cancer.
• Radiotracers target tumor cells, microenvironments, and metastases, offering precision in diagnosis and personalized treatment.
• Advances in radiopharmaceuticals integrate diagnostic and therapeutic applications, using radionuclides.
• Nanoparticles offer promising potential in theranostics, combining imaging and treatment in oncology.

## Graphical Abstract

**Figure:**
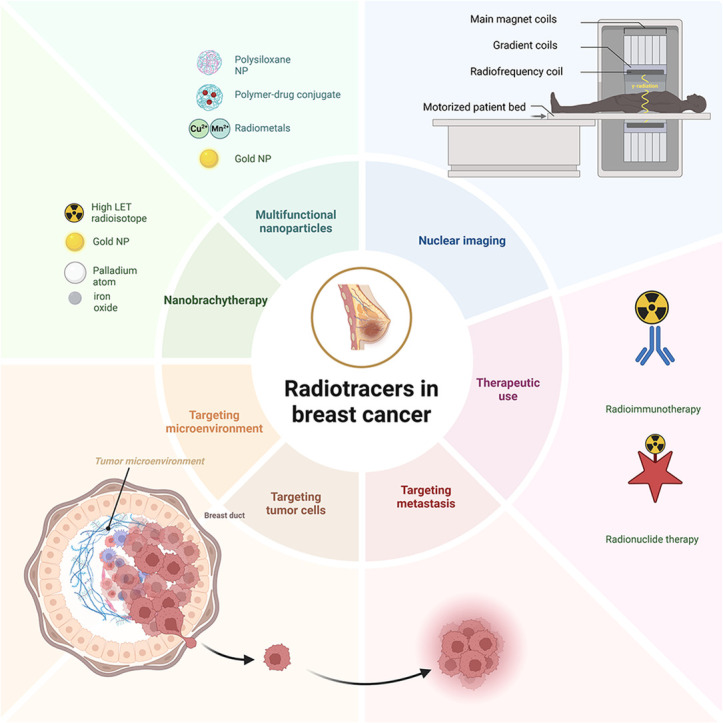

## Introduction

Radiolabeled molecules are essential in diagnosing, staging, monitoring therapy, and treating cancers. These molecules, composed of various radioligands, are administered in the body and emit various type of radiations, enabling effective therapy and real-time progress monitoring. Internal vectorised radiotherapies are becoming increasingly promising, particularly for heterogeneous cancers such as breast cancer (BC), where personalized treatment is crucial due to variable antigen expression. From 2010 to 2019, breast cancer incidence increased by 0.5 % annually, primarily affecting hormone-dependent cases [1]. Currently, BC is the most diagnosed cancer in women, accounting for 23 % of all cancer cases among women worldwide [2]. While treatment has reduced mortality, 30 % of patients experience relapse and metastasis.

BC is a heterogeneous disease classified into subtypes based on receptor expression: hormone-dependent cancers (ER+/PR+) in 70 % of cases, HER2-overexpressing cancers in 15 % of cases, and triple-negative breast cancer (TNBC) in 15 % of cases [2]. Various therapeutic strategies exist, including chemotherapy, targeted therapy, and hormone therapy, tailored to the cancer type and its stage.

Imaging stands as a cornerstone for evaluating treatment response, diagnosing, and assessing the extent of breast cancers. Nuclear medicine includes 2D imaging, such as scintigraphy, and 3D imaging, such as PET or SPECT scans (Positron Emission Tomography or Single-Photon Emission Computed Tomography) [3].

Through the injection or inhalation of a positron-emitting tracer, followed by positron emission and a reaction of annihilation with a nearby electron, two photons are produced. The PET technique relies on detecting radioactivity levels in the body, representing the local concentration of the tracer, and enables functional 3D imaging of several physiological or metabolic processes. The radioactive isotopes used include radioactive halogens (¹⁸F, ⁷⁷^,^⁷⁶Br, ¹²⁴I), ¹¹C, and radioactive metals (⁶⁸Ga, ⁶⁴Cu).

Radioactive halogens and carbon are commonly employed to label various radiopharmaceuticals, being more suitable for small molecules than radioactive metals. Conversely, radioactive metals are widely used to label larger molecules such as peptides and antibodies via the conjugation of metal chelators, such as 6-hydrazinonicotinic acid (HYNIC), diethylenetriaminepentaacetic acid (DTPA), 1,4,7-triazacyclononane-N,N′,N′′-triacetic acid (NOTA), and 1,4,7,10-tetraazacyclododecane-1,4,7,10-tetraacetic acid (DOTA).

On the other hand, single-photon emission computed tomography (SPECT) and scintigraphy use gamma-emitting radioisotopes such as ^99m^Tc, ^123^I, ^131^I, ^111^In, ^67^Ga, ^201^Tl, ^81m^Kr, and ^133^Xe. While PET offers the advantages of higher resolution and sensitivity, SPECT is more accessible and cost-effective. This difference arises due to the type of radioactivity (β⁺ or γ) used, which determines scanner designs. PET uses a ring of detectors allowing quasi-simultaneous detection by two individual detectors of the photons emitted after positron annihilation, while SPECT uses a planar detector or a ring of detectors to collect single gamma rays individually (Table 1).

**Table 1:** Comparison between SPECT and PET.

SPECT imaging	PET imaging
Cheapest	More expensive
Uses gamma emitting radioisotopes	Uses positron emitting radioisotopes
Less sensitive and resolution	Better sensitivity and resolution
One large crystal-based detector (for the more common design)	Ring of multiple detector

Both techniques provide quantitative information on radiotracer uptake using the standardized uptake value (SUV), calculated as follows:

SUV = $\frac{r}{\left(\frac{a^{\prime }}{w}\right)}$ where r is the radioactivity concentration (kBq/ml) measured within the region of interest, a′ is the decay-corrected activity of the radiolabeled compound injected (kBq), and w is the patient's weight (g).

SUV measures the accumulation of radiotracers in a tissue or tumor. Comparing radiotracer uptake in tumor tissue with that in healthy tissue provides essential information for determining tumor status or an individual's response to therapy. In addition, SUV thresholding is a technique used to subtract radioligand uptake in healthy tissue, enabling abnormal radiotracer accumulation to be observed.

Radiotracers have a wide range of uses as they can either serve as substrates for physiological pathways (such as [¹⁸F]-FDG), or target molecules with specific expression in cancer cells due to their abnormal physiology. Radiotracers possess specific characteristics, such as subnanomolar or nanomolar affinity for their targets, high specificity and/or selectivity, good plasma clearance combined with low non-specific plasma protein binding properties, and low toxicity. Their neutral or hydrophilic nature facilitates their subsequent elimination from the body, thus reducing radiation exposure. It is crucial for radiotracers to exhibit limited or well-characterized metabolism, as excessive or unpredictable metabolic breakdown can compromise their ability to target specific regions accurately. By ensuring stability or enabling precise measurement of metabolites, radiotracers allow for reliable kinetic modeling, which is essential for interpreting their distribution, target binding, and overall effectiveness in both diagnostic imaging and therapeutic applications.

To meet these requirements, the radiolabeling step involves the introduction of a radioisotope (Table 2), guaranteeing minimal alteration of the molecule's pharmacokinetics while conferring the desired radioactive properties. Molecules of interest can be monitored by SPECT or PET after replacement of an atom (or group of atoms) by a radioactive isotope, or by functionalization with a radioactive metal complex. Radioisotopes compatible with diagnostic radiopharmaceuticals (DRPs) are gamma-ray emitters, either directly (^111^In, ^123^I or ^99m^Tc), or following positron emission (^68^Ga, ^124^I).

**Table 2:** Radioisotopes commonly used for radiolabeling step in SPECT and PET imaging.

Radioisotopes	Element	Half-life	Units	Emission
^11^C	Carbon 11	20,37	min	Beta +
^18^F	Fluorine 18	1,82	hr	Beta +
^64^Cu	Copper 64	12,7	hr	Beta +
^68^Ga	Gallium 68	67,8	min	Beta +
^86^Y	Yttrium 86	14,7	hr	Beta +, Xray
^89^Zr	Zirconium 89	3,3	day	Beta +
^90^Y	Yttrium 90	2,67	day	Beta -
^99m^Tc	Technetium 99m	6	hr	Gamma
^111^In	Indium 111	2,8	day	Gamma, Xray, electron
^123^I	Iodine 123	13,2	hr	Gamma
^124^I	Iodine 124	4,18	day	Beta +, gamma
^125^I	Iodine 125	59,4	day	Auger
^131^I	Iodine 131	8	day	Gamma, Auger, Beta -
^177^Lu	Lutetium 177	6,65	day	Beta -
^225^Ac	Actinium 225	10	day	Alpha

Despite advancements in breast cancer diagnosis and treatment, challenges like tumor heterogeneity and therapy resistance persist, underscoring the need for innovative approaches. Radiotracers, with their capacity to non-invasively assess tumor biology and microenvironment dynamics, offer a promising solution. This review highlights recent progress in radiotracers for breast cancer imaging, focusing on their role in targeting the tumor microenvironment, addressing molecular heterogeneity, and advancing theranostic applications. These developments hold significant potential to refine precision medicine, enhance diagnostic accuracy, and improve therapeutic outcomes, paving the way for better integration of imaging and treatment in breast cancer management.

## Targeting tumor Cells

Understanding tumor cell metabolism is essential for diagnosis and treatment. Radiotracers play an essential role in this process by enabling the visualization and characterization of specific biological processes within tumors. These radiolabeled molecules can target various aspects of tumor biology, such as metabolism, proliferation, and the expression of specific marker, providing valuable informations on tumor progression, therapeutic response, and prognosis.

A fundamental aspect of tumor biology concerns the altered metabolism of cancer cells, characterized by increased nutrient uptake and utilization to sustain exacerbated proliferation.

One of the most commonly used radiotracers is [¹⁸F]-fluorodeoxyglucose ([¹⁸F]-FDG), a glucose analog. Tumor cells, which have high metabolic demands, avidly take up this radiotracer compared to healthy cells [4].

In addition to their high glucose requirements, tumor cells also need increased quantities of amino acids for DNA synthesis. For instance, thymidine kinase 1 (TK1), an enzyme involved in DNA synthesis, exhibits high activity in tumor cells, leading to increased incorporation of thymidine.

This metabolic feature is well-exploited in positron emission tomography (PET) imaging with [¹⁸F]-fluorothymidine ([¹⁸F]-FLT), a radiotracer that specifically targets proliferating tumor cells [5,6]. Such imaging techniques make it possible not only to detect primary tumors but also to monitor therapy response and identify metastatic extensions.

Furthermore, [¹⁸F]-fluciclovine, a leucine analog primarily utilized in prostate cancer, has demonstrated promising accumulation in breast cancer cells, offering valuable insights into neoadjuvant treatment responses [7].

Choline, a crucial component of cell membranes, also plays a significant role in tumor development by contributing to phosphatidylcholine synthesis. Choline consumption is elevated in cancer cells due to their abnormal proliferation. Radiotracers such as [¹⁸F]-fluorocholine and [¹¹C]-choline have demonstrated accumulation in breast carcinoma cells [[8], [9], [10]].

Beyond metabolic alterations, tumor cells are characterized by the abnormal expression of specific proteins, which can serve as potential targets for radiotracer-based imaging. Radiotracers targeting urokinase-type plasminogen activator receptor (uPAR), poly-ADP ribose polymerase (PARP), epidermal growth factor receptor (EGFR), and somatostatin receptor subtype 2 (SST2) have provided significant insights.

For example:

• Anti-uPAR 2G10-[¹¹¹In] specifically targets uPAR.
• PARP-targeting radiotracers such as [¹²⁴I]-I2-PARPi and [¹³¹I]-I2-PARPi are under study [11].
• [⁸⁹Zr]-Nimotuzumab, a high-affinity monoclonal antibody, targets EGFR.
• [⁶⁸Ga]-DOTATATE, an agonist radiotracer, targets SST2 [12].

These radiotracers provide crucial insights into breast cancer biology and potential therapeutic strategies, particularly in understanding tumor heterogeneity and improving treatment outcomes.

Deciphering breast cancer heterogeneity necessitates the use of specific radiotracers. For HER2-positive cancers, radiotracers like [⁸⁹Zr]-trastuzumab and [⁶⁴Cu]-DOTA-trastuzumab are tailored for detecting HER2-positive tumors. Additionally, [⁸⁹Zr]-pertuzumab has been employed to assess chemotherapy response in anti-HER2 antibody imaging [13].

[¹⁸F]-FES (16α-[¹⁸F]-fluoro-17β-estradiol) is a PET radiotracer used to evaluate estrogen receptor (ER) expression in breast cancer. Since ER plays a critical role in ER+ breast cancer cases, [¹⁸F]-FES imaging provides valuable insights into ER status across different tumor sites within the body. This whole-body imaging technique is particularly beneficial in clinical scenarios where metastasis biopsies are unsafe or impractical as well as in cases of suspected ER heterogeneity among tumor lesions. [¹⁸F]-FES PET can help resolve clinical dilemmas by accurately determining the extent of ER-positive disease, guiding treatment decisions, and improving patient management [14].

In triple-negative breast cancer (TNBC), unique molecular signatures necessitate particular radiotracers [15,16]. In some TNBC, PSMA expression can be effectively targeted by [⁶⁸Ga]-PSMA-ligand [17] for metastasis localization and potential anti-PSMA therapy. Additionally, for TNBC, overexpressed markers such as tissue factor, gpNMB protein, and folate receptor are targeted by radiotracers such as [⁶⁴Cu]-NOTA-ALT-836-Fab, [⁸⁹Zr]-DFO-CR011, and [⁸⁹Zr]-M9346A, respectively [18,19].

Moreover, smaller radiotracer-based agents, such as affibodies like ABY-025 [20] and HER2-Nanobody, offer advantages in imaging and monitoring breast cancer. Similarly, targeted agents such as NM600 and fluorthanatrace ([¹⁸F]-FFT) [21] cater to specific molecular aberrations, such as lipid raft targeting and PARP-1 overexpression in TNBC.

In summary, radiotracers represent a versatile toolset for interrogating the complex biology of breast cancer. By targeting various metabolic pathways, receptor expression patterns, and molecular signatures, these imaging agents provide valuable insights into tumor biology, enabling more accurate diagnosis, personalized treatment strategies, and improved patient outcomes.

## Targeting tumor microenvironment

In breast cancer imaging, the tumor microenvironment (TME) serves as a critical focus due to its pivotal role in tumor progression, therapeutic resistance, and metastatic potential. Radiotracers targeting the TME provide valuable insights into various facets of tumor biology, including hypoxia, angiogenesis, metabolic reprogramming, immune modulation, and extracellular interactions. These tracers enable non-invasive visualization of tumor dynamics, aiding in diagnosis, prognosis, and treatment strategies.

### Hypoxia imaging

Hypoxia is a feature of aggressive breast cancers, most often associated to therapy resistance and poor outcomes. Radiotracers such as [¹⁸F]-FMISO (fluoromisonidazole) and [¹⁸F]-HX4 (flortanidazole) [22] target hypoxic regions by accumulating in cells with low oxygen levels. [¹⁸F]-FMISO, the earliest nitroimidazole-based tracer, provides information on hypoxic severity, though it suffers from slow clearance and inconsistent correlation with hypoxia-inducible factor-1α (HIF-1α). The more advanced [¹⁸F]-HX4 offers improved clearance and stronger correlation with hypoxia markers, enhancing its utility in imaging hypoxic areas [22].

[¹⁸F]-FAZA (fluoroazomycin arabinoside) is another hypoxia-specific radiotracer that demonstrates higher hydrophilicity than [¹⁸F]-FMISO, resulting in faster clearance and lower background signals [23].

Copper-based radiotracers, including [⁶⁴Cu]-ATSM (copper-diacetyl-bis(N4-methylthiosemicarbazone)) and [⁶⁴Cu]-PTSM (copper-pyruvaldehyde-bis(N4-methylthiosemicarbazone)), add a theranostic dimension to hypoxia imaging. These tracers selectively accumulate in hypoxic regions via the reduction of Cu(II) to Cu(I). In addition to diagnostic imaging, they emit Auger electrons, inducing localized DNA damage in hypoxic cells. Similarly, ionic [⁶⁴Cu]CuCl₂ leverages the upregulation of copper transporter CTR-1 in hypoxic environments for precise targeting. These copper tracers not only visualize hypoxia but also demonstrate potential for therapy, particularly in targeting resistant cancer stem cells within hypoxic niches [23].

The presence of specific cell surface antigens within breast tumors offers unique opportunities for hypoxia targeted imaging. Hypoxia-regulated carbonic anhydrase IX (CAIX), a prevalent antigen in various cancers, including breast cancer, serves as a prime target. This protein is upregulated in the hypoxic microenvironment in response to pH variation within tumor cells. Radiopharmaceuticals like [⁸⁹Zr]-DFO-girentuximab and [⁸⁹Zr]-girentuximab [24] are tailored to target CAIX, facilitating precise PET imaging and enhancing diagnostic accuracy.

### Acidosis and metabolism

The acidic microenvironment of tumors, driven by lactate production and pH alterations, provides another imaging target. [¹⁸F]-ML-10 (2-(5-fluoro-pentyl)-2-methyl-malonic acid) detects extracellular acidification [25], while [¹¹C]-bicarbonate monitors pH variations. These tracers provide a window into the metabolic shifts accompanying apoptosis and therapy-induced tumor changes.

Advanced metabolic tracers such as [¹¹C]-lactate [26] or [¹⁸F]-lactate (3-[¹⁸F]-fluoro-2-hydroxypropanoic acid) and [¹⁸F]-FACH focus on lactate production and transport, highlighting glycolysis-driven metabolism in triple-negative breast cancer (TNBC). [¹⁸F]-FACH targets monocarboxylate transporters (MCT1 and MCT4), which are crucial for lactate export and survival in hypoxic and glycolytic tumor regions. Imaging with these tracers reveals metabolic heterogeneity and identifies aggressive tumor phenotypes [27].

The radiotracer [¹⁸F]-FSPG ([¹⁸F]-fluoropropyl-glutamic acid) represents another promising diagnostic tool, leveraging tumor-specific metabolic pathways for imaging. This tracer targets the xCT transporter, a glutamate-cystine exchanger associated with oxidative stress regulation and tumor progression. Breast cancers, particularly aggressive subtypes, overexpress xCT transporters, making them ideal targets for [¹⁸F]-FSPG imaging [28].

The pH (Low) Insertion Peptide (pHLIP) technology offers a promising strategy to exploit the acidic TME by delivering imaging and therapeutic agents selectively to cancer cells and tumor-associated macrophages (TAMs). When encountering an acidic pH (6.0–6.5), characteristic of the TME, protonation of specific carboxylate groups within the peptide sequence occurs. This increases hydrophobicity and triggers the peptide's insertion into the cell membrane, forming a stable transmembrane alpha-helix. The N-terminal end of the peptide remains extracellular, making it suitable for attaching imaging or therapeutic cargo, while the C-terminal end inserts into the membrane. Radiolabeled pHLIP peptides, such as those conjugated with fluorine-18 [¹⁸F], gallium-68 [⁶⁸Ga], or zirconium-89 [⁸⁹Zr], have demonstrated high tumor specificity and retention in preclinical models [29].

The tracer [^⁹⁹m^Tc]-HMPAO, driven by its intracellular interaction with glutathione (GSH), is another promising agent. GSH, which protects cells from oxidative stress, is involved in cancer resistance to chemotherapy and radiotherapy. GSH levels often vary depending on the breast cancer subtype (e.g., hormone receptor-positive, HER2-positive, or TNBC). TNBC, known for its aggressive behaviour and resistance to conventional therapies, may particularly benefit from GSH-targeted approaches. Imaging GSH with these tracers could help identify tumors that require combined GSH depletion and chemotherapy [30].

### Extracellular matrix and stromal targets

The extracellular matrix (ECM) and stromal cells play critical roles in tumor invasion and metastasis. Cancer-associated fibroblasts (CAFs), identified by fibroblast activation protein (FAP), are targeted by radiotracers like [¹²⁵I]-FAPI-01/[⁶⁸Ga]-FAPI-02 [31] and [^⁹⁹m^Tc]-FL-L3 [32], which offer insights into fibroblast dynamics, paving the way for both therapeutic interventions and imaging advancements.

Additionally, matrix metalloproteinases (MMPs), particularly MMP2 and MMP9, facilitate ECM degradation and invasion. [⁶⁸Ga]-NOTA-MMP-2/9 enables imaging of gelatinase activity, offering insights into metastatic potential, especially in invasive subtypes like TNBC [33].

Radiotracers targeting ECM-associated molecules also include PEGylated FUD peptides labeled with [⁶⁴Cu], which bind fibronectin (FN) [34], a key ECM protein involved in tumor growth and metastasis. Imaging FN dynamics with these tracers reveals tumor remodeling processes, aiding in therapeutic assessment and stratification of aggressive tumors.

### Angiogenesis and vascular dynamics

Angiogenesis is a cornerstone of tumor progression, supporting growth and metastasis. [⁶⁸Ga]-NOTA-RGD targets αvβ3 integrins, which are central to new blood vessel formation, enabling the visualization of angiogenesis and therapeutic responses. VEGF, a key angiogenic driver, is targeted by [⁸⁹Zr]-bevacizumab [35] and its receptor named VEGFR by specific tracers such as (R)-[¹¹C]-PAQ [36] and [⁶⁴Cu]-DOTA-VEGF. These tracers elucidate vascular remodeling and provide actionable data for anti-angiogenic treatments, particularly in TNBC, where vascular dynamics often influence therapeutic outcomes.

### Immune modulation and TAM imaging

Immune checkpoint inhibitors have revolutionized cancer therapy, and radiotracers targeting immune modulators such as PD-1 and PD-L1 are critical for assessing immunotherapy responses. Radiotracers like [⁸⁹Zr]-atezolizumab [37] and [⁸⁹Zr]-envafolimab [38] visualize PD-L1 expression, revealing immune escape mechanisms that tumors employ to evade the immune system.

Tumor-associated macrophages (TAMs), key mediators of immune suppression, are another critical target within the tumor microenvironment. Radiotracers such as [^⁹⁹m^Tc]-tilmanocept [39] and nanoparticle-based tracers like [⁸⁹Zr]-AI-HDL [40] are employed to image TAM distribution. These tracers provide valuable insights into the role of TAMs in tumor progression and highlight their potential as therapeutic targets.

### Cell death imaging

Cell death imaging focuses on detecting apoptosis and necrosis within the tumor microenvironment. Radiotracers such as [¹⁸F]-annexin V bind to phosphatidylserine, which becomes externalized on the cell membrane during apoptosis. Similarly, [^⁹⁹m^Tc]-duramycin targets phosphatidylethanolamine with high specificity, offering another method for visualizing apoptotic cells. Other probes focus on intracellular processes associated with apoptosis.

For instance:

• Caspase-targeting radiotracers like [¹⁸F]CP18 detect executioner enzymes active in apoptosis.
• [¹⁸F]-FBnTP highlights mitochondrial membrane potential dissipation during apoptotic signaling.

While these tracers effectively visualize apoptosis, transient signalling and specificity remain challenges requiring further optimization.

Radiotracers targeting loss of plasma membrane integrity, such as [⁶⁸Ga]-CDI, provide another means of detecting cell death. These tracers enter cells with compromised membranes and bind cytosolic proteins. [⁶⁸Ga]-CDI has shown favorable imaging characteristics, including rapid renal clearance, low uptake in normal tissues, and promising results in early human trials. It has potential for imaging tumor cell death across a range of cancers, including breast cancer, particularly when assessing responses to chemotherapy and radiotherapy [41].

### Exosome imaging

Exosomes and extracellular vesicles (EVs) facilitate intercellular communication within the tumor microenvironment (TME), playing a significant role in tumor progression and metastasis. Radiolabeled exosomes, tagged with isotopes such as [⁸⁹Zr] or [^⁹⁹m^Tc]-HMPAO (hexamethylpropyleneamine oxime), allow *in vivo* tracking of exosome distribution and uptake.

These radiolabeled exosomes enable the study of EV-mediated signaling pathways and their potential as biomarkers or therapeutic delivery systems. Radiolabeling techniques include:

1. **Surface labeling**, which uses radiometal chelators like NOTA and DOTA to attach isotopes to the exosome surface.
2. **Intraluminal labeling**, which employs ionophores like oxinate to incorporate isotopes into the exosome cargo.

Both methods offer unique advantages, but the choice of labeling strategy significantly impacts imaging results and functional interpretation [42].

### Theranostic perspectives

Theranostic radiotracers combine diagnostic imaging with therapeutic potential, representing a significant advancement in personalized oncology. For example:

• [⁶⁸Ga]-FAPI and [⁶⁴Cu]-ATSM are diagnostic agents that can pair with therapeutic isotopes like [¹⁷⁷Lu] or [²²⁵Ac] for targeted radionuclide therapy.

These dual-purpose agents exemplify the integration of imaging and treatment, enabling precise diagnosis and targeted intervention within a single framework.

Radiotracers targeting the tumor microenvironment are revolutionizing breast cancer imaging by addressing critical factors such as hypoxia, metabolism, immune modulation, extracellular matrix dynamics, and angiogenesis. Their therapeutic function lies in delivering targeted radionuclide therapy by pairing diagnostic radiotracers with therapeutic isotopes, enabling precise tumor eradication while minimizing damage to healthy tissues. This unique theranostic potential significantly enhances their value, providing integrated tools that simultaneously diagnose and treat disease.

As research progresses, these innovations are expected to refine precision medicine, improve clinical decision-making, and enhance outcomes for breast cancer patients.

## Targeting metastasis

In cancer management, identifying and characterizing metastases is crucial for determining disease prognosis and guiding treatment strategies. Traditionally, the detection of bone metastases has relied on techniques such as scintigraphy using [^⁹⁹m^Tc]-bisphosphonates for bone scans or [¹⁸F]-fluoride ([¹⁸F]-NaF) PET imaging [43]. Tracers commonly used for diagnosing tumors, such as [¹⁸F]-FDG, can also detect skeletal metastases alongside non-skeletal malignant lesions.

Progress in imaging technology have introduced more precise and efficient detection methods. For instance, positron emission tomography imaging with radiotracers like [⁶⁸Ga]-NO2AP-BP [22], derived from bisphosphonates labeled with gallium-68, offers enhanced sensitivity and spatial resolution compared to traditional methods.

## Therapeutic use

Innovations in cancer treatment continue to evolve, with researchers exploring novel therapeutic strategies to target cancer cells more effectively while minimizing damage to healthy tissues. Among these approaches, radionuclide therapy (RT) and radiopharmaceutical therapy (RPT), also called internal vectorized radiation therapy, have emerged as promising modalities. These therapies deliver focused doses of radiation directly to tumor sites.

### Radionuclide and radiopharmaceutical therapy

Radiation therapy typically involves ionizing radiation administered externally to destroy cancer cells. In contrast, internal radiation therapy, or brachytherapy, involves placing radioactive sources directly inside or near the tumor for more localized treatment. RT represents a distinct approach by using systemically administered radionuclides that concentrate specifically in tumors. This concept is further enhanced in RPT, which combines radionuclides with compounds that specifically target cancer cells, increasing precision and efficacy.

Internal vectorized radiation therapy uses vectors, such as antibodies, peptides, ligands, or nanoparticles, to deliver radioactive substances directly to cancer cells. These vectors are designed to target specific markers on cancer cells, such as overexpressed receptors or antigens. Once the vector-radionuclide combination is administered intravenously, orally, or directly into the tumor cavity, the radionuclides emit radiation, destroying cancer cells from within while sparing surrounding healthy tissues.

### Ionizing radiation

Three types of ionizing radiation are commonly used: photons (X-rays and γ-rays), electrons, and alpha particles. Photons are useful for follow-up imaging, as they penetrate tissues and can be detected for diagnostic purposes. For therapeutic applications, electrons and alpha particles are preferred. Alpha particles exhibit higher linear energy transfer and a shorter range, while beta particles have lower linear energy transfer and a longer range. Radiotherapy particles generate free radicals that break covalent bonds in water molecules, causing DNA damage through double-strand breakage.

### Examples of radiopharmaceutical agents

RPT and RT represent cutting-edge techniques for delivering focused radiation directly to cancer cells. Chelated radionuclides such as ¹⁷⁷Lutetium and ¹⁵³Samarium [44] provide a personalized approach to cancer treatment, minimizing systemic toxicity while enhancing therapeutic efficacy.

Specific agents demonstrate the potential for targeted treatment in breast cancer:

• [¹⁷⁷Lu]-DOTATATE targets SSTR2 (somatostatin receptor 2) [45].
• [¹⁷⁷Lu]-FAPI-02 targets fibroblast activation protein (FAP) [46].

### Therapeutic applications in TNBC

Internal vectorized radiation therapy can also be used to treat TNBC. For instance:

• Lipid rafts are targeted using [¹⁷⁷Lu]-NM600 [47], an alkylphosphocholine analog.
• Angiogenesis is targeted with [¹⁷⁷Lu]-DOTA-diZD [48], involving the therapeutic agent ZD7474 (vandetanib), which has high affinity for VEGFR (vascular endothelial growth factor receptor).

Auger radiation emitters, such as [¹³¹I]-MAPI [49] (Meitner Auger PARP inhibitor) and [¹²⁵I]-PARPi-01 [50], show promise for targeting cancer due to their ability to cause lethal DNA damage in close proximity to binding sites. These agents are particularly effective in BRCA-mutated TNBC (BReast CAncer susceptibility genes), offering a highly localized treatment approach with minimal systemic effects.

### Radioimmunotherapy

Radioimmunotherapy combines antibodies with radionuclides to provide a targeted and precise approach to cancer treatment and imaging. By attaching radioactive tags to antibodies, these molecules can locate and deliver therapeutic or imaging payloads to cancer cells or disease-associated proteins. This approach enables accurate imaging, localized radiation therapy, and real-time monitoring of treatment response.

Radiolabeled antibodies contribute to personalized medicine, reducing side effects by minimizing damage to healthy tissues [51]. Examples include:

• PAN-622, which targets human aspartyl asparaginyl β-hydroxylase (HAAH) [52].
• CSPG4-specific monoclonal antibodies (mAbs) radiolabeled with ¹¹¹In or ²¹³Bi [53].

A clinical trial involving the radiolabeled antibody BAY 86-1903 is ongoing (NCT03507452). BAY 86-1903, a mesothelin-targeted antibody conjugated with ²²⁷Th (thorium-227), enables the delivery of alpha particles to cancer cells [54].

### Targeted alpha therapy (TAT)

TAT, a specific form of TRT, utilizes alpha-particle-emitting radionuclides, such as ²²⁵Ac (actinium-225), to selectively destroy cancer cells. For hormone receptor-positive breast cancer, delivery systems like hu11B6, which targets human kallikrein 2 (hK2), have shown potential for highly specific and effective treatment [55].

### Radiolabeled chemotherapy

Radiolabeled chemotherapy integrates traditional chemotherapeutic agents with radioactive substances to enhance targeted cancer therapy. For example:

• Antibiotics like doxorubicin and idarubicin, radiolabeled with isotopes such as ⁸⁶Re or ⁹⁰Y, offer dual-modality treatment by combining cytotoxic effects with localized radiation therapy.
• Paclitaxel has been conjugated with glucuronic acid and radiolabeled with ¹³¹I [56] or incorporated into a nano-system ([¹³¹I]-HSA-PTX) using human serum albumin pre-labeled with ¹³¹I.
• GRPR-positive breast cancer can be targeted using paclitaxel delivered via [¹⁷⁷Lu]-bombesin radiotherapy [57].

For HER2+ breast cancer, the radiotherapeutic agent [¹¹¹In]-NLS-trastuzumab exhibits excellent nuclear localization, effectively killing HER2+ cells and preventing resistance to treatment [58].

## Radioactive nanoparticles

The combination of multiple imaging modalities or the integration of imaging with remotely controlled therapeutic activity offers great potential to improve diagnosis, image-guided therapy, and post-treatment monitoring [59]. To achieve these goals, multifunctional nanoparticles have emerged as a more efficient solution than traditional molecules. A variety of nanoparticles can be easily synthesized by manipulating the chemical composition of their core and shell, as well as their size, shape, and structure.

Multifunctional nanoparticles exhibit better biodistribution and pharmacokinetics than conventional molecules. For example, their size can be optimized to prevent diffusion into healthy tissues while increasing preferential accumulation in solid tumors via the enhanced permeability and retention (EPR) effect, which also prolongs tumor retention time. These properties make multifunctional nanoparticles highly attractive for tumor imaging and cancer therapy guided by imaging [60]. However, their clinical application depends not only on efficacy but also on their capacity for elimination from the body.

Compared to the injected dose, the amount of nanoparticles reaching tumors is generally low after intravenous administration [61]. To ensure safe elimination, nanoparticles must be designed appropriately. Ultrasmall nanoparticles (with a hydrodynamic diameter of less than 6–10 nm, depending on surface chemistry) are generally suitable for renal clearance, preserving nanoparticle integrity and limiting the dissemination of potentially toxic fragments [62]. Larger nanoparticles must be biodegradable to avoid long-term accumulation in the body.

### Nanoparticles as carriers or active agents

Two strategies have been developed to exploit the potential of nanoparticles for imaging and therapy:

1. **Nanoparticles as carriers of active agents.**
2. **Nanoparticles as active agents themselves.**

These approaches are exemplified by the clinical application of radiolabeled albumin nanoparticles (HSA) and radioactive rhenium sulfide nanoparticles [63,64].

Radiolabeled HSA nanoparticles are prepared prior to administration by adding pertechnetate in the presence of tin(II) ions to HSA nanoparticles (95 % of HSA particles are smaller than 80 nm). These nanoparticles are clinically applied for detecting sentinel lymph nodes (SLNs) in various cancers. For breast cancer, administration depends on tumor location. An activity of 200 MBq is administered in several fractions (5–20 MBq each) via intradermal, subcutaneous, or peri-areolar injection for superficial tumors, while intra- or peri-tumoral injection is performed for deep tumors.

Radioactive rhenium sulfide nanoparticles are indicated for the treatment of inflammatory arthritis in adults. They are used during inflammatory flare-ups in the joints of the shoulder, elbow, wrist, ankle, and hip following intra-articular injection. Unlike radiolabeled HSA nanoparticles, rhenium nanoparticles are intrinsically radioactive, as part of their rhenium atoms are radioactive (¹⁸⁶Re).

Recent research has focused on developing radioactive multifunctional nanoparticles for imaging and treating breast and other cancers [65]. Three main applications of radioactive nanoparticles have been identified:

1. Studying biodistribution and pharmacokinetics.
2. Acting as radiotracers for diagnosis.
3. Treating tumors via high linear energy transfer (LET) particles emitted during radionuclide decay (nanobrachytherapy and radionuclide therapy).

### Nuclear imaging for biodistribution studies

Multifunctional nanoparticles hold great potential for imaging and therapeutic applications, prompting studies on their distribution, accumulation in tissues, elimination, vascular residence time, and retention time in tumors. Nuclear imaging techniques like SPECT and PET are particularly attractive for these studies due to their high sensitivity and quantitative capabilities [66].

Since most nanoparticles are not intrinsically radioactive, radionuclides are incorporated into them either by replacing components with radioactive isotopes, neutron activation, or radiolabeling. Radiolabeling can be achieved by substituting specific atoms or groups (e.g., -OH replaced with ¹⁸F or ¹²⁴/¹²⁵/¹³¹I) or by forming stable complexes with radiometals. A challenge with this approach is that altering the chemical properties of nanoparticles may change their biodistribution and pharmacokinetics. However, the high sensitivity of nuclear imaging allows the use of minimal amounts of radioisotopes, ensuring only minor modifications to the nanoparticles.

Decorating nanoparticles with linear or macrocyclic polyaminocarboxylate chelators is an effective strategy for medical imaging. These chelators form stable complexes with gadolinium ions for MRI and radiometals for SPECT (¹¹¹In) and PET (⁶⁴Cu, ⁶⁸Ga, ⁸⁹Zr) [67,68]. Radiolabeled gold nanoparticles (Au@DTDTPA, Au@TADOTAGA) and polysiloxane nanoparticles (AGuIX®) decorated with linear diethylenetriaminepentaacetic acid (DTPA) derivatives or DOTAGA-like macrocycles have shown safe behavior in preclinical models. After intravenous injection into healthy mice, these ultrasmall nanoparticles circulate freely without accumulating in healthy tissues, and most are eliminated via renal clearance [[69], [70], [71]].

Dalong Ni, Weibo Cai, and colleagues demonstrated that PET imaging could monitor the biodistribution and tumor accumulation of ultrasmall porous silica nanoparticles functionalized with DOTA-like macrocycles in mice bearing subcutaneous 4T1 tumors [72].

### Multimodal imaging with nanoparticles

Nanoparticles can also be designed to combine different imaging modalities, allowing the collection of complementary data. For example, Wiesner and colleagues developed fluorescent and radioactive silica nanoparticles for medical imaging [[67], [73], [74]]. Fluorescence is achieved through the covalent grafting of organic dyes to the polysiloxane network, while radioactivity is introduced via radioiodination. These nanoparticles, functionalized for targeting integrins, show promise for SLN localization and staging during biopsy procedures. They also hold potential for image-guided surgery.

The combination of PET and MRI is particularly attractive for medical imaging, as it leverages the high sensitivity of PET with the high spatial resolution of MRI [68,75]. Iron oxide nanoparticles, known for their superparamagnetic properties, have been central to PET/MR imaging development. Immobilizing positron emitters onto these nanoparticles makes them suitable as contrast agents for MRI and radiotracers for PET [76,77].

AGuIX® nanoparticles, composed of polyaminocarboxylate chelators, can also form stable complexes with gadolinium ions for MRI and positron-emitting radiometals such as ⁶⁴Cu and ⁶⁸Ga for PET imaging [78].

### Nanobrachytherapy

Brachytherapy uses sealed short-range radiation sources for treating solid tumors, such as cervical, prostate, breast, esophageal, and skin cancers [79]. Implanting a sealed radioactive source in or near the tumor delivers localized radiation, minimizing side effects compared to external beam therapy. However, this approach is invasive.

Nanobrachytherapy addresses these limitations by using intratumoral injection of nanoparticles composed of high LET radionuclides. Most high LET radionuclides (e.g., ¹⁷⁷Lu, ²²⁵Ac) form stable complexes with macrocyclic polyaminocarboxylate chelators. These nanoparticles typically have an inorganic core (e.g., gold or iron oxide) coated with high LET radionuclide chelates [80,81].

Marc-André Fortin and colleagues proposed alternative designs, including gold-core nanoparticles containing radioactive gold atoms (¹⁹⁸Au) or gold-core nanoparticles encapsulated in an inorganic shell composed of radioactive palladium atoms (¹⁰³Pd) [82].

## Conclusion

The integration of radiolabeled molecules into diagnostic and therapeutic approaches marks a significant milestone in cancer research and clinical practice. These molecules, as radiotracers or therapeutic agents, offer exceptional specificity by targeting cancer cells, the tumor microenvironment, and metastases.

In breast cancer, radiopharmaceuticals play a vital role in imaging and treatment, utilizing advanced modalities like PET and SPECT to visualize tumor metabolism, receptor expression, and microenvironmental dynamics. Radiotracers targeting markers such as HER2, ER, and hypoxia-regulated proteins exemplify the impact of personalized oncology. Therapeutic radiopharmaceuticals, particularly with alpha and beta -emitters, enable localized tumor destruction with minimal impact on healthy tissues. Theranostic agents, combining diagnostic and therapeutic functions, represent a breakthrough in integrating imaging and therapy.

Nanotechnology further enhances radiopharmaceutical potential, offering multifunctional platforms for imaging, therapy, and controlled biodistribution. Radioactive nanoparticles provide innovative solutions for overcoming challenges in cancer management, enabling precision targeting and safe elimination.

Challenges persist, including limited tumor uptake and the complexity of design. Future efforts should optimize pharmacokinetics, biodistribution, and efficacy. Collaboration among molecular biology, nanotechnology, and nuclear medicine is essential to advance these tools.

In conclusion, radiopharmaceuticals are transforming breast cancer diagnosis and therapy, combining precision targeting with advanced imaging and therapeutic modalities. Innovation and interdisciplinary efforts will ensure their full potential is realized, improving outcomes for patients with this complex disease.

## CRediT authorship contribution statement

**Chloé Jean:** Writing – review & editing, Writing – original draft, Conceptualization. **Stéphane Roux:** Writing – review & editing, Writing – original draft, Conceptualization. **Abdelilah Aziz:** Writing – review & editing. **Maxence Mocquery-corre:** Writing – review & editing. **Rana Bazzi:** Writing – review & editing. **Yacine Merrouche:** Writing – review & editing. **Stéphane Dedieu:** Writing – review & editing. **Nicolas Etique:** Writing – review & editing. **Dimitri Papathanassiou:** Writing – review & editing. **Jérôme Devy:** Writing – review & editing, Writing – original draft, Supervision, Conceptualization.

## Declaration of competing interest

The authors declare that they have no known competing financial interests or personal relationships that couId have appeared to influence the work reported in this paper.
